# Functional Adaptation of Oromotor Functions and Aging: A Focused Review of the Evidence From Brain Neuroimaging Research

**DOI:** 10.3389/fnagi.2019.00354

**Published:** 2020-01-09

**Authors:** Chia-Shu Lin

**Affiliations:** ^1^Department of Dentistry, School of Dentistry, National Yang-Ming University, Taipei, Taiwan; ^2^Institute of Brain Science, School of Medicine, National Yang-Ming University, Taipei, Taiwan; ^3^Brain Research Center, National Yang-Ming University, Taipei, Taiwan

**Keywords:** aging, mastication, swallowing, adaptation, brain plasticity, motor learning

## Abstract

“Practice makes perfect” is a principle widely applied when one is acquiring a new sensorimotor skill to cope with challenges from a new environment. In terms of oral healthcare, the traditional view holds that restoring decayed structures is one of the primary aims of treatment. This assumes that the patient’s oromotor functions would be recovered back to normal levels after the restoration. However, in older patients, such a structural–functional coupling after dental treatment shows a great degree of individual variations. For example, after prosthodontic treatment, some patients would adapt themselves quickly to the new dentures, while others would not. In this Focused Review, I argue that the functional aspects of adaptation—which would be predominantly associated with the brain mechanisms of cognitive processing and motor learning—play a critical role in the individual differences in the adaptive behaviors of oromotor functions. This thesis is critical to geriatric oral healthcare since the variation in the capacity of cognitive processing and motor learning is critically associated with aging. In this review, (a) the association between aging and the brain-stomatognathic axis will be introduced; (b) the brain mechanisms underlying the association between aging, compensatory behavior, and motor learning will be briefly summarized; (c) the neuroimaging evidence that suggests the role of cognitive processing and motor learning in oromotor functions will be summarized, and critically, the brain mechanisms underlying mastication and swallowing in older people will be discussed; and (d) based on the current knowledge, an experimental framework for investigating the association between aging and the functional adaptation of oromotor functions will be proposed. Finally, I will comment on the practical implications of this framework and postulate questions open for future research.

## Introduction

Pierre Fauchard, known as the father of modern dentistry, wrote in his classic textbook *The Surgeon Dentist, a Treatise on Teeth* that teeth are the primary object of dental therapy (Fauchard, 1946). At that time, dental treatment would follow a relatively simple logic: because most of the oral diseases related the teeth and oral cavity, as Fauchard’s textbook has focused (Lynch et al., 2006), the primary aim of dental treatment is to manage the patients’ teeth. However, in the modern days, the stomatognathic system is considered as an assembly composed of ‘the mouth, teeth, jaws, pharynx, and related structures as they relate to mastication, deglutition, and speech’ (PubMed^1^). The modern view holds that not only the individual anatomical components (e.g., the teeth or the tongue) but also the interaction between these components would play a key role in oral functions.

From the view of functional physiology, one may place a greater emphasis on the functions of the stomatognathic system, i.e., focusing on how the individuals improve their functional performance when facing environmental stress (Frisancho, 1993). Based on this view, a critical challenge of managing sensorimotor disorders is whether the patients adapt themselves to a new oral condition. In dental practice, for example, installing a new denture may not necessarily improve the patients’ satisfaction of oromotor functions (Carlsson and Omar, 2010) or significantly improve their nutritional status (Toniazzo et al., 2018). It should be noted that restoring the anatomical deficits would still be the primary aim of dental treatment. However, the notion that oral sensorimotor functions would be fully regained, as long as anatomical deficits are well-restored, may be oversimplified. In terms of oral functions, what is ignored here is the role of functional adaptation, which generally refers to ‘the process whereby the organism has attained a beneficial adjustment to the environment’ (Frisancho, 1993). As the saying goes, ‘practice makes perfect’: the issues about how individuals acquire new oral sensorimotor skills, and the mechanisms underlying the individual difference of adaptation, require further investigation.

In this review, I will focus on individual differences in oral sensorimotor functions. I argue that functional adaptation is associated with the brain mechanisms of cognitive processing and motor learning and that these variations in brain signatures of cognitive processing and motor learning may underlie the individual differences in oral sensorimotor adaption. The brain-stomatognathic mechanisms underlying sensorimotor adaptation may provide important insight into the age-related changes in oral functions and contribute to the clinical management of geriatric patients. The article will be organized into four sections:

(1) The age-related changes in the stomatognathic system will be recapitulated, and the concept of the ‘brain-stomatognathic axis’ (BSA) will be defined. The association between aging and the individual differences in the BSA will be highlighted.
(2) The general framework concerning the association between aging, adaptation, and compensation will be reviewed. I will focus on the concept of ‘motor learning’ and explain why it plays a key role in sensorimotor adaptation in older people. The brain mechanisms of motor learning from recent neuroimaging evidence will be discussed.
(3) Recent neuroimaging findings [primarily based on magnetic resonance imaging (MRI)] regarding aging and the brain mechanism of mastication and swallowing will be systematically discussed. Specifically, the role of functional adaptation and the individual differences in the oromotor functions will be highlighted.
(4) Finally, a research model for oral sensorimotor adaptation will be proposed. I will focus on using neuroimaging methods to quantify the individual differences in compensatory mechanisms in older people. Further investigation may provide important insight into the age-related changes in oral functions and contribute to the clinical management of geriatric patients.

## Aging And the Brain-Stomatognathic Axis

### Aging and the Coupling Between Brain Structure and Functions

Broadly defined as ‘the gradual irreversible changes in structure and function of an organism that occur as a result of the passage of time’ (PubMed^1^), the effect of aging is associated with both structural and functional changes. However, it does not necessarily mean that aging has the same effect on all the structural and functional aspects. In terms of the cognitive abilities of elderly people, the decline of perceptual speed is a life-long change, gradually decreasing by year; in contrast, the decline of verbal memory is a late-life change, i.e., occurring in the later periods of life (Hedden and Gabrieli, 2004). In terms of the variations in brain morphology, as age increases, the brain does not change in size homogenously. For example, the prefrontal cortex and the medial temporal lobe (where the hippocampus resides) show a more pronounced decrease in size (Curiati et al., 2009; Lemaitre et al., 2012), but the primary sensory cortices (e.g., the visual cortex and the somatosensory cortex) are less sensitive to the aging effect (Lemaitre et al., 2012). Notably, there is a critical coupling between functional and structural variations. The size of the prefrontal cortex and perceptual speed, which is associated with prefrontal functions (Muller-Oehring et al., 2013), showed a similar age-related trend. The size of the hippocampus and verbal memory, which is associated with hippocampal functions, also showed a similar age-related trend (Hackert et al., 2002). The structural–functional coupling suggests that in elderly people, variations in brain signatures are critically related to individual differences in mental functions.

### Age-Related Changes in the Stomatognathic System: A View From the Brain-Stomatognathic Axis

As age increased, the individuals showed significant changes in several biomechanical features of the stomatognathic system (for a detailed review, please see Avivi-Arber and Sessle, 2018). In general, these changes included a decrease in the maximal bite force (Ikebe et al., 2005), the maximum tongue pressure (Utanohara et al., 2008), and the rate of oral diadochokinesis (Ben-David and Icht, 2018). Similar to the age-related changes in the brain, the changes in oral functions are also coupled with structural alterations. For example, the age-related reduction in tongue pressure may be associated with the decreased fiber size of the intrinsic tongue muscle (Cullins and Connor, 2017). An age-related increase in sensory threshold was also identified, including an increased threshold in thermal pain, touch, and two-point discrimination in the orofacial regions (Heft and Robinson, 2010). An age-related decrease in the intrafusal fibers of muscle spindles, a critical proprioceptor for sensory feedback from the jaw-closing muscles, may account for the decreased masticatory functions (Winarakwong et al., 2004). In general, these age-related changes in structure and biomechanical features contributed to worse masticatory performance (Morita et al., 2018).

It should be noted that while the biomechanical features showed an overall age-related reduction, these changes did not fully account for the individual variations in the oral sensorimotor performance of the older people. The results collected from a local community in Taipei, Taiwan revealed a heterogeneous effect of aging: while some parameters showed a significant age-related decline (e.g., oral mixing ability, the efficiency of saliva swallowing) (Lin et al., 2017a, 2019), others did not (e.g., number of masticatory cycles, unstimulated salivary flow rate, and masseter muscle volume) (Lin et al., 2017a, 2019). Notably, the stomatognathic parameters *per se* may not fully explain the individual differences in a certain function. For example, more saliva does not necessarily reflect a higher swallowing frequency (Persson et al., 2018; Lin et al., 2019), and the number of chewing strokes before swallowing was not significantly associated with masticatory performance (Fontijn-Tekamp et al., 2004). Therefore, regarding the individual differences in oral sensorimotor functions, one may consider the contribution from features other than the stomatognathic system. Since aging has a profound effect on the brain (Curiati et al., 2009; Lemaitre et al., 2012), variations in brain signatures, including brain morphology and intrinsic connectivity, should be considered when one interprets the individual differences in oral sensorimotor functions.

### The Brain-Stomatognathic Axis Focuses on Individual Differences in Age-Related Changes

The concept of the BSA has been proposed to emphasize that when explaining the individual differences in oral sensorimotor functions, one needs to consider the brain and the stomatognathic system as a closely coupled assembly. Moreover, the BSA should be considered a complex adaptive system so that feeding behaviors can dynamically respond to environmental changes (Holland, 1982). At the conceptual level, research on the BSA is different from research on the brain mechanisms underlying oral sensorimotor functions, which have been gradually unraveled at the cause-effect level, thanks to the systematic investigation of animal research (Sessle, 2006; Avivi-Arber and Sessle, 2018). The BSA is rooted in the background of the neuroanatomical infrastructure of the stomatognathic system, but it focuses on how the brain and the stomatognathic system, as a whole, respond to challenges in feeding behaviors. Moreover, the BSA highlights the individual differences in such a behavioral adaptation.

There are several practical reasons why the issues of the BSA need to be highlighted. First, many geriatric disorders have posed great challenges in dental practice, including dysphagia (Rommel and Hamdy, 2016), stroke (Schimmel et al., 2017) and dementia (Daly et al., 2018), and most of these disorders are associated with the disruption in the brain functions. Second, since the BSA focused on unraveling the individual differences in oral sensorimotor functions, the framework would be particularly suitable in diagnosis and outcome evaluation of oral sensorimotor functions, which has posed a great challenge in clinical practice, e.g., assessing patients with dementia for pain (Delwel et al., 2019) and masticatory ability (Weijenberg et al., 2019). Third, from the experimental perspective, the advent of neuroimaging techniques has made it feasible to quantify individual brain signatures based on a large sample size (Dubois and Adolphs, 2016). Neuroimaging research has proven useful in unraveling the brain mechanisms underlying the individual variations of sensorimotor functions, which I will elaborate in the following section. In general, the focus on the BSA will meet the increasing demand for dental researchers to translate research findings to clinical practice, especially for managing geriatric and special needs patients.

## Aging and Adaptations in Motor Action

### The Theoretical Framework of Adaptation, Reserve, and Compensation

In terms of geriatric medicine, a discrepancy may exist between one’s anatomical condition (structure) and the actual performance (function). For example, in patients with Alzheimer’s disease, why do some people behave better than others? A critical underlying factor is the individual differences in ‘reserve’—the variation in brain signatures and cognitive experience—which would underlie the “differential susceptibility to functional impairment” in the presence of a disorder (Barulli and Stern, 2013). For example, an increased brain size and a rich life experience of cognitive ability are associated with a lesser chance for elderly patients to develop severe disabilities (for detailed reviews, see Barulli and Stern, 2013 and Cabeza et al., 2018). Critically, these brain and cognitive reserves are associated with individual differences in compensation, a notion that interprets how functional status is maintained under an (anatomically) aging status (Cabeza et al., 2018). The compensatory mechanisms are crucial in explaining how people adapt to the environment. Through compensation, individuals can functionally adapt to a changing environment by recruiting neural resources (Cabeza et al., 2018), rather than drastically modifying the anatomical apparatus. Such an adaptive coupling between the brain and behavior, primarily based on brain plasticity (Pascual-Leone et al., 2005), is the key to the BSA being regarded as a complex adaptive system (Holland, 1982).

### Adaptation of the Stomatognathic Structure

In order to cope with the environmental stress, the adaptation from the functional aspects (i.e., the brain functions) and from the structural aspects (i.e., the alterations in the peripheral anatomical device) would play a key role. The age-related changes in the masseter, the primary jaw-closing muscle in the stomatognathic system, would demonstrate the adaptation of the stomatognathic structure. Evidence from clinical research revealed that the increased age was associated with the delayed latency in muscle reflex (Peddireddy et al., 2006) and lower maximal isometric voluntary contraction in elderly women (Gaszynska et al., 2017). The structural and functional features of the masseter also related to systemic factors. For example, masseter thickness was positively correlated with grip force (Yamaguchi et al., 2018). Notably, these general decreases in biomechanical features occurred in the individual with normal aging. However, not all the structural and functional features showed the consistent age-related ‘degradation’ or ‘degeneration.’ For example, the age-related change in the size of the masseter and its muscle fibers was not pronounced, evidenced by both human and animal research (Norton et al., 2001; Lin et al., 2017a; Daboul et al., 2018). Evidence from animal research revealed that the older subjects showed more nerve terminal branches at the neuromuscular junction of the masseter, compared to the younger subjects (Elkerdany and Fahim, 1993). As the age increased, the masseter may show the plasticity or remodeling at the neuromuscular junction (Elkerdany and Fahim, 1993). In terms of oral rehabilitation, the adaptive process can also be examined by assessing masticatory performance and muscle work, which showed an initial decrease after denture installation, and then a full recovery back to the original status (Eberhard et al., 2018). The human masseter may be less vulnerable to the age-related degeneration, due to its major role in feeding behavior (Avivi-Arber and Sessle, 2018).

### Motor Control, Motor Learning, and ‘Relearning’

The control of an action, which is predominantly mandated by the primary motor cortex, is associated with complicated cognitive processing, such as memory and choice-making (Ebbesen and Brecht, 2017). Motor control is generally defined as ‘the process of transforming sensory inputs into consequent motor outputs’ (Wolpert et al., 2001) and motor learning is about the process of refining this association, in order to adapt sensorimotor transformations for environmental challenges (Wolpert et al., 2001). Behind these ‘transformations’ is the complicated mechanism of building a predictive model that can bidirectionally match a motor command and the corresponding sensory outcomes. Under such a computational framework, motor learning can be regarded as a process of acquiring such a forward/inverse model (Wolpert et al., 1998, 2001). Critically, the model can be reshaped to respond to environmental changes so that the performance can be dynamically improved (Wolpert et al., 2001). This association between learning and oromotor functions is manifested in patients who wear a new set of dentures. While the stomatognathic structure is restored (e.g., by replacing the missing teeth with prosthesis), patients need to learn how to chew with the new dentures. From the computational view of brain functions, the individuals need to relearn this chewing action, i.e., building a new model of sensorimotor transformation, in contrast to the old one (i.e., their experience of chewing without dentures).

What are the brain correlates associated with motor learning? When people learn a new motor skill, the prefrontal cortex, the secondary motor area, and the cerebellum show distinct activation (Lage et al., 2015). The prefrontal cortex is critical to cognitive processing, such as attending to a movement, switching from one movement to another, and monitoring of the progression of a movement (for a detailed review on the prefrontal functions, see Ridderinkhof et al., 2004). The secondary motor area consists of the supplementary motor area and the premotor cortex (Marvel et al., 2019). While the activation of the primary motor cortex is associated with the execution of movement, the activation of the secondary motor area is associated with planning or preparation of movement (Marvel et al., 2019), which can be guided by external cues or by memory (Heuninckx et al., 2010). As shown in the following section, cumulating evidence has consistently revealed that older people engage an extended brain region when learning a new motor skill, including the prefrontal area, the supplementary area, and the premotor cortex. The pattern of brain activation may be associated with an increased cognitive effort, e.g., a greater demand for multisensory integration and attentional control during movement (Ward, 2006; Seidler et al., 2010). The cerebellum is a critical component in forming the predictive model of motor learning (Wolpert et al., 1998). It receives the error between the actual and the anticipated action and gives a new motor command that is corrected for the error message (Wolpert et al., 1998). The cerebellum integrates and fine-tunes sensory and motor information for refining the models so that movement can be automatically performed (Ramnani, 2014; Schmahmann, 2019). Compared to the basal ganglia, the cerebellum plays a dominant role in sensorimotor adaptation, i.e., modulating motor commands on the basis of sensory feedback, via error-based learning (Bostan and Strick, 2018). Furthermore, the cerebellum has an extensive connection with the prefrontal and the parietal lobes as well as the motor areas (Stoodley, 2012). The age-related changes in cerebellar morphology may be associated with both motor and cognitive declines in older people (Bernard and Seidler, 2014; Schmahmann, 2019).

### Age-Related Changes in the Brain Signatures of Motor Learning

Evidence from neuroimaging research has revealed that older people, compared to younger people, showed greater activation of the primary and secondary motor areas, the cerebellum, and the prefrontal cortex when they are acquiring a new motor skill (Mattay et al., 2002; Heuninckx et al., 2005; Wu and Hallett, 2005; Rowe et al., 2006). This age-related activation is associated with increased task complexity (Heuninckx et al., 2005) and reduced response time of a motor task (Mattay et al., 2002). The findings suggest that when coping with a more challenging environment (i.e., a difficult motor task), older people would compensate for their performance by recruiting more extended brain regions beyond the primary motor cortex (Ward, 2006). This difference in brain mechanisms may reflect a decreased automaticity in older people, i.e., performing one motor task without being interfered with by another task (Ward, 2006). Comparing brain activation before and after motor learning, younger, but not older, people showed reduced activation of the cerebellum, a region critically related to automaticity of movement (Wu and Hallett, 2005). During the retention of a learned skill, older subjects showed a smaller deactivation of the frontal lobe (Berghuis et al., 2019). The extended engagement with the prefrontal areas implied that the older subjects demanded more cognitive efforts in learning a skill.

Importantly, the age-related changes in the brain mechanisms of motor learning were manifested not only in regional activation but also in the interregional connections. Aging is related to increased local effective connectivity within the motor network, centered at the premotor cortex (Rowe et al., 2006). In the older subjects, better motor performance was associated with an increased resting-state functional connectivity between the cerebellum and the primary and secondary motor areas (Seidler et al., 2015). Similarly, an increased connectivity between the primary motor cortex and the premotor/prefrontal cortex was associated with a faster psychomotor speed (Michely et al., 2018). The local connectivity efficiency of the primary somatosensory and motor cortices was correlated with gait stability in older, but not younger, subjects (Di Scala et al., 2019). The findings suggest that the increased performance in older people is associated with a greater role of premotor and prefrontal areas. Moreover, at the scale of the whole-brain connectome, older people showed a stronger, not weaker, connection between the prefrontal cortex and the sensorimotor module of the orofacial part (Chan et al., 2014). Decreased segregation in brain networks plays a key role in age-related declines in motor performance (King et al., 2018). The findings suggest that the age-related difference in the architecture of the functional connectivity of the brain may be associated with individual differences in motor performance.

## Motor Learning and Aging: Research Evidence From Mastication and Swallowing

### Brain Mechanisms of Mastication

For decades, the brain mechanisms of mastication have been systematically investigated, primarily via animal research (for detailed reviews, see Sessle, 2006; Avivi-Arber and Sessle, 2018). The following sections will focus on recent findings based on neuroimaging methods, primarily based on the MRI. As a non-invasive brain imaging method, functional MRI has identified several brain regions associated with mastication that have been consistently reported by animal research (Lin, 2018). Moreover, neuroimaging findings have revealed more complicated mechanisms underlying the adaptation of oromotor functions.

Recent functional MRI findings of mastication in older people are summarized in Table 1. One of the earliest neuroimaging studies on human mastication was performed by Onozuka and colleagues, who asked subjects to chew gum during the MRI scan (Onozuka et al., 2002). The study consistently identified an increased activation of the primary somatosensory and motor cortices, which plays a key role in motor control and has also been found in animal research (Avivi-Arber and Sessle, 2018). The activation at the somatosensory region, which was also found during the adaptation of facial tactile stimuli (Custead et al., 2017) and stimulation of periodontal ligament (Trulsson et al., 2010), especially highlighted the role of sensory feedback in mastication. Interestingly, pronounced activation was also found in the supplementary motor area and the cerebellum (Onozuka et al., 2002), and an increased functional connectivity between the motor areas and the cerebellum was found during chewing (Quintero et al., 2013b). Moreover, the prefrontal activation was found only during jaw movement, but not during hand movement, in the older subjects (Fang et al., 2005). Further studies revealed that the primary motor cortex was dominantly engaged when a chewing block was initiated or terminated (Quintero et al., 2013a). While activation of the primary motor cortex was identified in both the younger and the older subjects, activation of the prefrontal cortex was more pronounced in the older than the younger subjects (Onozuka et al., 2003). The increased functional connectivity between the motor areas and the prefrontal cortex was also reported (Quintero et al., 2013b). Consistently, imaging meta-analysis also revealed a common coactivation of the primary somatosensory/motor cortex, the secondary motor area, the prefrontal cortex, and the cerebellum (Lin, 2018). The findings revealed that beyond the primary somatosensory and motor cortices, an extended network of cognitive processing and motor learning is critical to chewing.

**TABLE 1:** Results of literature search of the recent neuroimaging findings (from 2000 January 1 to present) of aging and oromotor functions.

**ID**	**Source**	**Subjects (disease/treatment)**	**Age (year) (mean or range)**	**Imaging methods**	**Major findings**
**(A) Summary of recent neuroimaging findings of aging and masticatory functions^1^**					
1	Lin et al., 2015	Healthy, OA	64.2	sMRI, rs-fMRI	‘… in the premotor cortex, a reduction of GMV and rsFC would reflect declined masticatory performance. The positive correlation between DLPFC connectivity and masticatory performance implies that masticatory ability is associated with cognitive function in the elderly’ (Lin et al., 2015).
2	Lin et al., 2017b	Healthy, OA vs. YA	64.4	rs-fMRI	‘… in OA, higher masticatory performance is associated with a widespread pattern of mastication-related hubs. Such a widespread engagement of multiple brain regions associated with the MPI may reflect an increased demand in sensorimotor integration, attentional control and monitoring for OA to maintain good mastication’ (Lin et al., 2017b).
3	Fang et al., 2005	Healthy, OA vs. YA	60–70	fMRI	‘For movements of the face (chewing, opening and closing of mouth), the prefrontal cortex was activated in the old age group but finger and hand movements never activated the prefrontal cortex in any age’ (Fang et al., 2005).
4	Onozuka et al., 2003	Healthy, OA vs. YA	65–73	fMRI	‘In all subjects, chewing resulted in a bilateral increase in the BOLD signals in the sensorimotor cortex, cerebellum, thalamus, supplementary motor area, and insula, and a unilateral increase in the right prefrontal area. In the first three regions, the signal increases were attenuated in an age-dependent manner, whereas, in the right prefrontal area, the converse was seen. The remaining two regions showed no significant differences with ages. These results indicate that chewing causes regional increases in neuronal activity in the brain, some of which are age-dependent’ (Onozuka et al., 2003).
5	Shoi et al., 2014	Prosthesis	66.1	fMRI	‘Brain activation during gum chewing with the full dental arch occurred in the middle frontal gyrus, primary sensorimotor cortex extending to the pre-central gyrus, supplementary motor area, putamen, insula, and cerebellum. However, middle frontal gyrus activation was not observed during gum chewing with the shortened dental arch. These results suggest that shortened dental arch affects human brain activity in the middle frontal gyrus during gum chewing, and the decreased middle frontal gyrus activation may be associated with decreased masticatory function’ (Shoi et al., 2014).
6	Luraschi et al., 2013	Prosthesis	70.3	fMRI	‘The right and the left precentral gyrus (PRCG) and post-central gyrus (POCG) were identified with significant activation across all three functional tasks. A statistically significant increase in the level of activity between T0 and T2 (POCG: *P* = 0.022; PRCG: *P* = 0.017) was found during jaw clenching tasks’ (Luraschi et al., 2013).
7	Kimoto et al., 2011	Prosthesis	64–79	fMRI	‘… the gum-chewing task in elderly edentulous patients resulted in differential neural activity in the frontal pole within the prefrontal cortex between the 2 prosthodontic therapies-mandibular CD and IOD’ (Kimoto et al., 2011).
8	Yan et al., 2008	Prosthesis	48–72	fMRI	‘Increased blood oxygen level dependent signals in the primary sensorimotor cortex were found in patients with implant-supported fixed dentures. Other activated areas included prefrontal cortex, Broca’s area, premotor cortex, supplementary motor area, superior temporal gyrus, insular, basal ganglion, and hippocampus… Activation of the primary sensorimotor cortex in patients with implant-supported dentures might explain the improved tactile, stereognostic ability, and mastication functions, which are more similar to the natural dentition’ (Yan et al., 2008).
9	Miyamoto et al., 2005	Prosthesis	56.9	Near-infrared optical topography	‘Results revealed a significantly (*P* < 0.001; paired *t*-test) increased cerebral regional blood volume during maximum voluntary clenching task by implant-retained prosthesis. There were no statistically significant differences between patients with and without prosthesis in the latency to the maximum regional blood volume after the task. Conclusively, clenching can be effective for increasing cerebral blood volume; accordingly maintenance of normal chewing might prevent the brain from degenerating’ (Miyamoto et al., 2005).
**(B) Summary of recent neuroimaging findings of aging and swallowing functions^2^**					
10	Humbert et al., 2011	Alzheimer’s disease	74.3	fMRI	‘Disease-related differences were evident where the AD group had significantly greater BOLD response in the insula/operculum than the old. These findings have significant clinical implications for control of swallowing across the age span and in neurodegenerative disease. Greater activation in the insula/operculum for the AD group supports previous studies where this region is associated with initiating swallowing. The AD group may have required more effort to “turn off” swallowing centers to reach the intentional swallowing off-state’ (Humbert et al., 2011).
11	Humbert et al., 2010	Alzheimer’s disease	74.3	fMRI	‘… the AD group had significantly lower Blood-Oxygen-Level-Dependent (BOLD) response in many cortical areas that are traditionally involved in normal swallowing (i.e., pre and post-central gyri, Rolandic and frontal opercula). There were no regions where the AD group showed more brain activity than the healthy controls during swallowing, and only 13% of all active voxels were unique to the AD group, even at this early stage. This suggests that the AD group is not recruiting new regions, nor are they compensating within regions that are active during swallowing’ (Humbert et al., 2010).
12	Lin et al., 2019	Healthy, OA	69.1	sMRI	‘In healthy older adults, swallowing efficiency was positively correlated with cerebellar GMV. The findings suggested that in older people, structural variations of the brain may play a key role in individual differences in swallowing performance’ (Lin et al., 2019).
13	Lowell et al., 2012	Healthy	52	fMRI	‘The greater connectivity from the left hemisphere insula to brain regions within and across hemispheres suggests that the insula is a primary integrative region for volitional swallowing in humans’ (Lowell et al., 2012).
14	Martin et al., 2007	Healthy	74.2	fMRI	‘Activation of the post-central gyrus was lateralized to the left hemisphere for saliva and water swallowing, consistent with our findings in young female subjects. Comparison of saliva and water swallowing revealed a fourfold increase in the brain volume activated by the water swallow compared to the saliva swallow, particularly within the right premotor and prefrontal cortex. This task-specific activation pattern may represent a compensatory response to the demands of the water swallow in the face of age-related diminution of oral sensorimotor function’ (Martin et al., 2007).
15	Windel et al., 2015	Healthy, OA vs. YA	64	fMRI	‘The results indicate that the highly automated swallowing network retains its functionality with age. However, seniors with higher SCR during swallowing appear to also engage areas involved in attention control and emotional regulation, possibly suggesting increased attention and emotional demands during task performance’ (Windel et al., 2015).
16	Malandraki et al., 2011	Healthy, OA vs. YA	70.2	fMRI	‘Both groups showed activations in the major motor areas involved in the initiation and execution of movement; however, areas involved in sensory processing, sensorimotor integration and/or motor coordination and control, showed reduced or limited activity in the elderly’ (Malandraki et al., 2011).
17	Teismann et al., 2010	Healthy, OA vs. YA	71.6	MEG	‘The main finding of this study was an increase of somatosensory cortical activation during swallowing execution in elderly subjects compared to the young control group. This effect was present in both hemispheres. These results point to adaptive cerebral changes in response to aging effects on the complex process of swallowing. Our finding underlines the relevance of age matched control groups in neuroimaging studies related to deglutition or other complex sensorimotor processes’ (Teismann et al., 2010).
18	Humbert et al., 2009	Healthy, OA vs. YA	72.3	fMRI	‘The group of older adults recruited more cortical regions than young adults, including the pericentral gyri and inferior frontal gyrus pars opercularis and pars triangularis (primarily right-sided). Saliva swallows elicited significantly higher BOLD responses in regions important for swallowing compared to water and barium…. These findings suggest that older adults without neurological insult elicit more cortical involvement to complete the same swallowing tasks as younger adults’ (Humbert et al., 2009).
19	Suntrup-Krueger et al., 2017	Stroke	73.7	CT/MRI	‘This study gives new insights on the cortical representation of single components of swallowing and airway protection behaviors. The lesion model may help to risk-stratify patients for dysphagia and pneumonia based on their brain scan’ (Suntrup-Krueger et al., 2017).
20	Galovic et al., 2017	Stroke	75.6	dMRI	‘… early swallowing recovery is influenced by white matter lesions disrupting thalamic and corticobulbar projection fibers. Late recovery is determined by specific cortical lesions affecting association fibers. This knowledge may help clinicians to identify patients at risk of prolonged swallowing problems that would benefit from enteral tube feeding’ (Galovic et al., 2017).
21	Mihai et al., 2016	Stroke	56.6	fMRI/dMRI	‘Overall, patients showed decreased fMRI-activation in the entire swallowing network apart from an increase of activation in the contralesional primary somatosensory cortex (S1). Moreover, fMRI activation in contralesional S1 correlated with initial dysphagia score. Finally, when lesions of the pyramidal tract were more severe, recovered swallowing appeared to be associated with asymmetric activation of the ipsilesional anterior cerebellum. Taken together, our data support a role for increased contralesional somatosensory resources and ipsilesional anterior cerebellum feed forward loops for recovered swallowing after dysphagia following stroke’ (Mihai et al., 2016).
22	Galovic et al., 2016	Stroke	71,76	MRI	‘Mild impairment of oral intake correlates with damage to a widespread operculo-insular swallowing network. However, specific lesions of the anterior insula lead to severe impairment and tube dependency and clinicians might consider early enteral tube feeding in these patients’ (Galovic et al., 2016).
23	Suntrup et al., 2015	Stroke	73.7	CT/MRI	‘In particular, right hemispheric lesions of the pre- and post-central gyri, opercular region, supramarginal gyrus and respective subcortical white matter tracts were related to dysphagia, with post-central lesions being especially associated with severe swallowing impairment…. Distinct brain lesion locations are related to the incidence, severity and pattern of swallowing dysfunction’ (Suntrup et al., 2015).
24	Li et al., 2014	Stroke	65.2	rs-fMRI, dMRI	Stroke patients with dysphagia exhibited dysfunctional connectivity mainly in the sensorimotor-insula-putamen circuits based on seed-based analysis of the left and right M1 and SMA and decreased connectivity in the bilateral swallowing-related ROIs functional connectivity network. Additionally, white matter tract connectivity analysis revealed that the mean fractional anisotropy of the white matter tract was significantly reduced, especially in the left-to-right SMA and in the corticospinal tract’ (Li et al., 2014).
25	Momosaki et al., 2012	Stroke	66.1	SPECT	‘The rCBF in Brodmann areas 4 and 24 was significantly lower in the dysphagia group. The highest area under the curve was found in Brodmann area 4. In this area, 80% sensitivity and 60% specificity for discriminating dysphagia were achieved with an optimal cutoff value. When analyzed with novel methods, SPECT imaging can be useful for predicting the risk of dysphagia and subsequent aspiration in post-stroke patients’ (Momosaki et al., 2012).
26	Teismann et al., 2011	Stroke	63.5	MEG	‘Our results demonstrate strong bilateral reduction of cortical swallowing activation in dysphagic patients with hemispheric stroke. In hemispheric stroke without dysphagia, bilateral activation was found. In the small group of patients with brainstem stroke we observed a reduction of cortical activation and a right hemispheric lateralization’ (Teismann et al., 2011).
27	Li et al., 2009	Stroke	70.9	fMRI	‘Cerebral activation during swallowing tasks was localized to the precentral, post-central and anterior cingulate gyri, insula and thalamus in all groups. Activation of volitional swallowing in dysphagic unilateral hemispheric stroke patients might require reorganization of the dominant hemispheric motor cortex, or a compensatory shift in activation to unaffected areas of the hemisphere’ (Li et al., 2009).

*^1^According to a PubMed-based search with the following combination of keywords: (aging OR age-related OR older OR elderly) AND (chew^∗^ OR masticat^∗^) AND (neuroimaging OR “brain imaging” OR MRI) AND brain, with the publication date from 2000/01/01. ^2^According to a PubMed-based search with the following combination of keywords: (aging OR age-related OR older OR elderly) AND (swallow^∗^ OR deglut^∗^) AND (neuroimaging OR “brain imaging” OR MRI) AND brain, with the publication date from 2000/01/01. CT, computed tomography; dMRI, diffusion magnetic resonance imaging; fMRI, functional magnetic resonance imaging; MEG, magnetoencephalography; OAs, older adults; sMRI, structural magnetic resonance imaging; SPECT, single photon emission computed tomography; rCBF, regional cerebral blood flow; rs, resting-state, structural magnetic resonance imaging; YA, younger adults.*

Notably, activation of the prefrontal cortex was frequently associated with activation of the secondary motor area, including the supplementary motor area and the premotor cortex. The supplementary motor area plays a pivotal role in preparation and planning of voluntary movements (Thickbroom et al., 2000) and the premotor cortex, together with the parietal lobe and the somatosensory area, is critical to the integration of polymodal motion processing with movement (Bremmer et al., 2001). The connectivity between the prefrontal cortex, the supplementary motor cortex, and the premotor cortex, is critical to attention to action (Rowe et al., 2002). The coactivation of these cognitive regions (i.e., the prefrontal cortex, the premotor cortex, and the supplementary motor area) has been identified not only on healthy subjects (Onozuka et al., 2002, 2003) but also on the patients receiving a denture, which may suggest an adaptive experience of using a denture (Yan et al., 2008; Kimoto et al., 2011; Shoi et al., 2014).

These functional MRI studies revealed the brain activation associated with the processing of mastication. Recent neuroimaging findings have revealed that individual differences in masticatory performance were associated with intrinsic brain signatures, such as gray matter volume and resting-state functional connectivity. In older subjects, the masticatory performance was positively correlated with gray matter volume of the premotor cortex and the lateral prefrontal cortex (Lin et al., 2015) and an increased connectivity between these motor areas and the cerebellum (Lin et al., 2015). Moreover, when investigating the association between functional connectivity and masticatory performance, one could identify a qualitatively different pattern: in the older subjects, those who had a higher chewing performance showed a stronger connectivity between the core sensorimotor regions and the non-primary areas (e.g., the prefrontal and the parietal areas and the insula) (Lin et al., 2017b). It is noteworthy that the prefrontal cortex is one of the brain regions that showed the greatest degree of age-related volumetric decline (Curiati et al., 2009; Lemaitre et al., 2012). Therefore, the findings suggest that in older people, in addition to the age-related decline in structure or biomechanical features (e.g., tooth loss or the decreased biting force), the individual variation in masticatory performance may be associated with brain functions of cognitive processing and learning.

### Brain Mechanisms of Swallowing

Much neuroimaging evidence regarding swallowing has been reported over the past decades. A synthesis from imaging meta-analysis revealed that water swallowing and saliva swallowing are associated with different patterns of brain activation (Soros et al., 2009). Water swallowing requires a higher degree of sensory-motor integration, which shows a higher activation at the parietal lobe. In contrast, saliva swallowing is more associated with the premotor areas, which are crucial for the initiation and control of movements (Soros et al., 2009). Notably, this pattern of brain activation revealed an age-related difference. The primary somatosensory cortex showed a lower activation in the older subjects, compared to the younger subjects (Malandraki et al., 2011). In the older subjects, water swallowing was engaged with stronger activation of the right premotor and prefrontal cortices compared to saliva swallowing (Martin et al., 2007). To complete the same swallowing tasks, the older subjects showed more cortical involvement as the younger subjects (Humbert et al., 2009). Another critical finding revealed that older subjects showed longer reaction times and higher skin conductance responses (SCRs) during swallowing (Windel et al., 2015). Importantly, a stronger SCR was associated with greater brain activation in areas related to sensorimotor and emotional processing, suggesting increased cognitive-affective regulation during task performance (Windel et al., 2015). In stroke patients with dysphagia, there was a distinct activation and lesion locations of the primary somatosensory and motor cortices (Li et al., 2009; Suntrup et al., 2015) and the insula (Galovic et al., 2016) and changes in the connection of these regions (Li et al., 2014). Furthermore, the pattern of brain activation differed substantially between healthy controls and the patients with cognitive impairment. During swallowing, the patients with Alzheimer’s disease showed a lower activation of the primary somatosensory and motor cortices and no recruitment of new brain regions, suggesting insufficient compensation (Humbert et al., 2010). In contrast, they showed a higher activation of the insula, when intentionally inhibit swallowing (Humbert et al., 2011). These findings from both healthy and disease groups revealed that in older people, swallowing is associated with the brain regions of cognitive processing and motor learning.

Notably, these neuroimaging findings were largely based on functional MRI, which investigated the swallowing-related brain signals by contrasting different task conditions (e.g., saliva swallows vs. resting). The relatively lower temporal resolution (by seconds) poses a limitation on experimental design and data interpretation of fMRI research. In contrast, the magnetoencephalography (MEG) study is superior in recording the brain signals at a higher temporal resolution (by milliseconds). It can be synchronized with other assessments, such as electromyography, for recording the brain signals associated at different stages of swallowing. In one study, the whole-brain MEG scan was associated with electromyography and revealed a bilateral increased somatosensory activation in the elderly subjects, compared to the younger controls (Teismann et al., 2010). Moreover, the same method revealed that during swallowing execution, the cortical activation was lower in the stroke patients with dysphagia vs. without dysphagia (Teismann et al., 2011). The findings extended the previous results from fMRI research, demonstrating the changes in brain activity synchronized with swallowing movement.

Recent neuroimaging evidence has also revealed that the gray matter volume of the posterior cerebellum was associated with an increased swallowing performance (Lin et al., 2019). Notably, part of the identified posterior cerebellum (the cerebellar crus and lobule VII) did not directly connect with the primary sensorimotor area but with the prefrontal cortex and the posterior parietal lobe (Schmahmann, 2019). Therefore, the findings suggest that swallowing performance may partly reflect individual variations in the cognitive control of swallowing. Notably, in the study, swallowing performance was quantified by the repetitive saliva swallowing task (RSST), a simple and safe test that represents the number of voluntary swallow in 30 s (Oguchi et al., 2000a, b). Recent findings from Sweden and Taiwan revealed that RSST scores were not significantly associated with the degree of saliva secretion (Persson et al., 2018; Lin et al., 2019). These findings together suggest that in older people, individual differences in swallowing performance may be attributed to variations in brain signatures, rather than the peripheral conditions (e.g., saliva secretion) *per se*.

## Functional Adaptation of Oromotor Skills – An Hypothetical Experimental Framework

### Functional Adaptation of Oromotor Skills: Why Do We Need More Evidence?

While the current neuroimaging evidence has provided a general picture of the brain signatures associated with the individual differences in the BSA, it is difficult to directly translate these research findings to clinical applications. From the perspective of clinical practice, a crucial question is to quantify the degree of individual differences and to provide a better prediction of the outcome of adaptation on an individual basis. The critical issues are to clarify (a) what functional performance is regained, (b) what structural impairment is compensated for, and (c) what brain regions (or networks) are associated with the individual differences in the adaptation. These goals can be achieved only with a valid experimental design. In the following section, I will propose three conditions of experimental design that may facilitate the design of a neuroimaging study about the functional adaptation of oromotor functions.

### Proposed Conditions for a Study on the Adaptation of Oromotor Functions

#### Quantify the Relationship Between Functional Performance and Challenges (Figure 1A)

**FIGURE 1:**
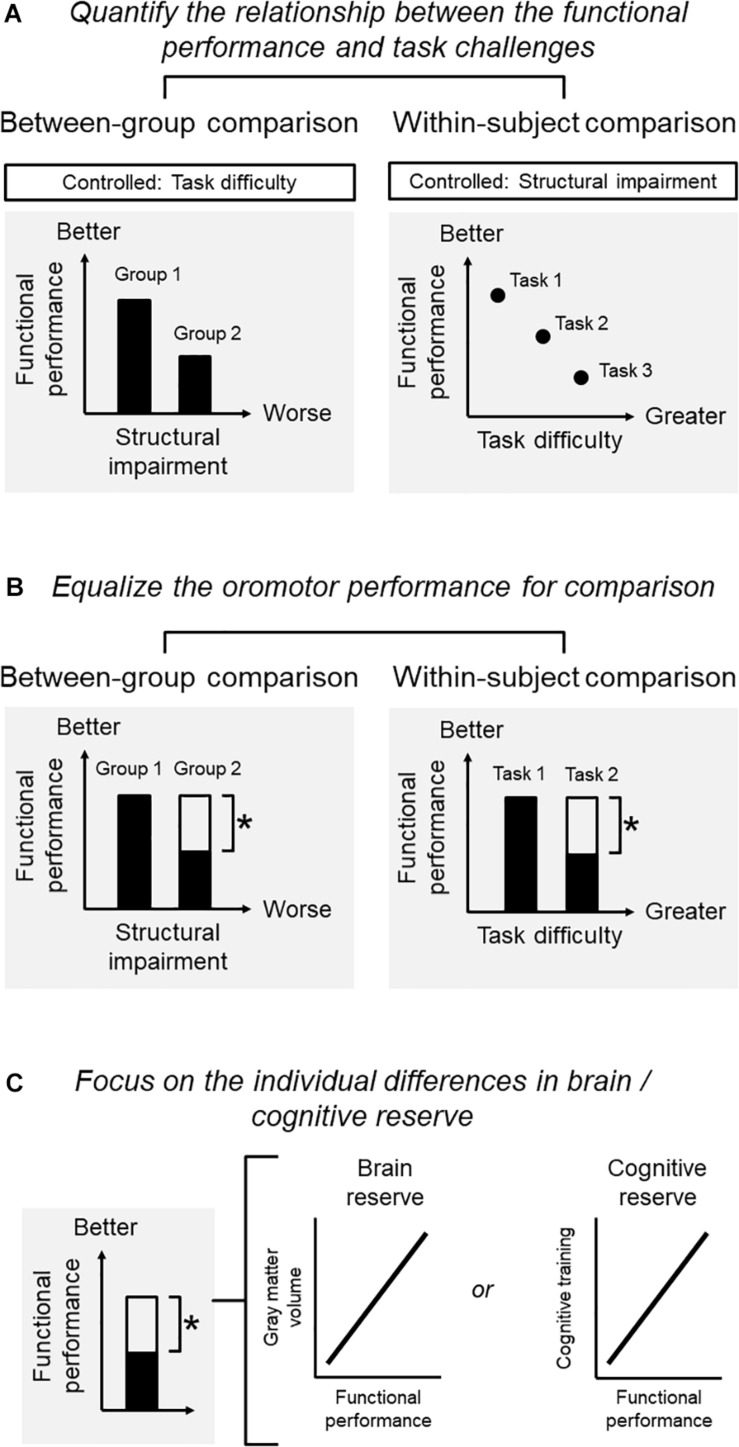
Conditions for a study on the adaptation of oromotor functions. **(A)** Quantify the relationship between functional performance and challenges: functional performance, structural impairment, and task difficulties may interact with each other so that one factor needs to be controlled when the other two are investigated. For example, when adopting a between-group comparison, one may be interested in the performance associated with structural impairment. The task for assessing the performance should be controlled. When adopting a within-subject comparison, one may be interested in the performance for the same subject under different levels of task difficulty. The degree of structural deficits should then be controlled. **(B)** Equalize the oromotor performance for comparison): for a within-subject design, it is critical to define the degree of functional adaptation that should ideally bring the individual back to the original level (before diseases) or, at least, to an acceptable level whereby the individual can perform daily functions satisfactorily. For a between-group comparison, one would focus on the contrast between two groups with different degrees of structural impairment, yet showing the same degree of functional performance. **(C)** Focus on the individual differences in brain/cognitive reserve: in older people, a sufficient brain and cognitive reserve would be necessary for them to develop better compensations against impairment increased performance. Without clarifying the brain-performance relationship, the identified changes in brain signatures merely represent an age-related phenomenon. One would expect that the degree of functional performance in the adapted group (i.e., the group with structural impairment yet showing an adequate performance) to be correlated with the brain signatures.

A primary condition is to quantify the relationship between functional performance, structural impairment, and task difficulties, depending on the purpose of the research. Notably, these factors may interact with each other so that one should control one of the factors when examining the association between the other two factors. For example, when adopting a between-group comparison, one may be interested in the masticatory performance associated with structural impairment. Here, the task difficulty for assessing masticatory performance should be controlled. When adopting a within-subject comparison, one may be interested in the performance for the same subject under different levels of task difficulty. The degree of structural impairment should then be controlled (Figure 1A).

#### Equalize the Oromotor Performance for Comparison (Figure 1B)

In older people, the ‘overactivation’ of some brain regions (e.g., the prefrontal recruitment during chewing) would indicate a compensatory process or ‘working harder’ than their younger counterparts (Reuter-Lorenz and Cappell, 2008). However, such an ‘overactivation’ may represent maladaptive neuroplasticity of sensorimotor functions, rather than an underlying compensation (Sessle, 2019). Therefore, the use of the term ‘compensation’ should be confined to situations where a substantial degree of functional performance was regained against the observed structural impairment. For a within-subject design, it is critical to define the degree of functional adaptation that should ideally bring the individual back to the original level (before diseases) or, at least, to an acceptable level whereby the individual can perform daily functions satisfactorily. The same principle applies to between-group comparisons (e.g., younger vs. older subjects). For example, a neuroimaging study reported that younger and older subjects showed different degrees of brain activation when consolidating acquired motor skills into memory. The specific changes in the older group could be interpreted as a compensatory mechanism for adapting their functions only when the two groups showed a similar degree of motor learning (e.g., the same learning rate) (Berghuis et al., 2019). Therefore, the between-group comparison would focus on the contrast between two groups with different degrees of structural impairment, yet showing the same degree of functional performance (Figure 1B).

#### Focus on the Individual Differences in Brain/Cognitive Reserve (Figure 1C)

As shown previously, in older people, a sufficient brain and cognitive reserve would be necessary for them to develop better compensations against impairment (Cabeza et al., 2018). Theoretically, to claim that the changes in brain signatures are associated with the compensation on a certain performance, one needs to identify the association between the changes of the brain and the increased performance. Without clarifying the brain-performance relationship, the identified changes in brain signatures merely represent an age-related phenomenon (Cabeza et al., 2018). Clinically, it would be more important to differentiate those who are capable of compensation from those who cannot, via an assessment of their brain/cognitive profiles. Since the individual capacity of compensation is associated with brain and cognitive reserve, one would expect that the degree of functional performance in the adapted group (i.e., the group with structural impairment yet showing an adequate performance) to be correlated with the brain signatures, e.g., gray matter volume or intrinsic connectivity. Notably, such a correlation holds only in the adapted group but not in the non-adapted group (i.e., the group with structural impairment and showing insufficient performance).

### Brain Mechanisms of Functional Adaptation of Mastication: An Example of Neuroimaging Research

To illustrate how the three proposed conditions are applied to clinical research, I will propose an example study about mastication. The aim of this research is to understand (a) the potential mechanisms that could explain why some individuals can maintain their masticatory performance, even if they have a poorer status of teeth contact, and (b) whether the identified brain signatures can predict individual differences in functional adaptation.

#### Quantify the Relationship Between Functional Performance and Challenges

**FIGURE 2:**
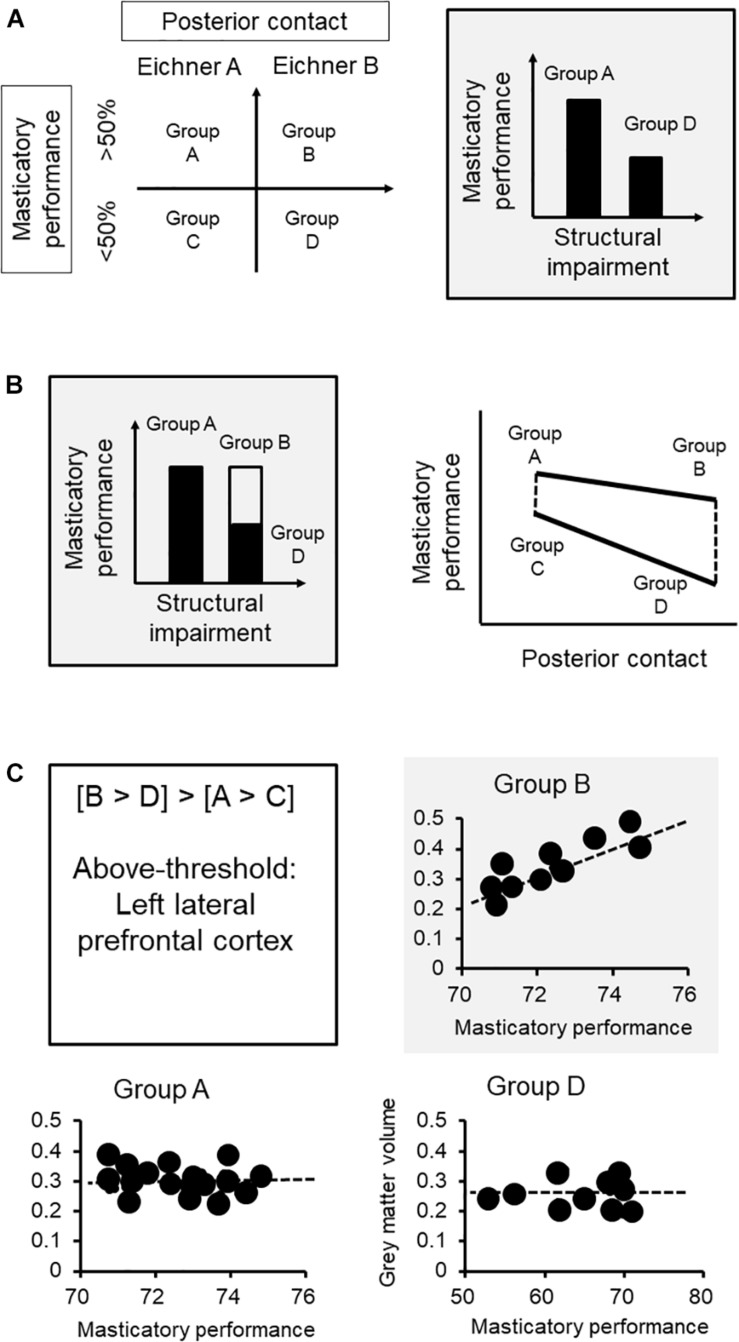
Brain mechanisms of functional adaptation of mastication: an example of neuroimaging research. **(A)** We first clarify the association between functional performance and challenges. The sample was subgrouped by the degree of structural impairment based on the Eichner class, which reflects the degree of posterior contact, and the functional aspect according to masticatory performance. **(B)** Each group was further subgrouped by masticatory performance via median split. Based on the subgrouping, subgroup B represents the subjects who had less teeth contact but maintained a good masticatory performance (i.e., the adapted group) that showed no statistically significant difference from subgroup A. Subgroup D, which represents the subjects who did not adapt to the loss of posterior contact, would showed a significantly lower masticatory performance, compared to either subgroup A or B. The interactional effect ([B vs. D] vs. [A vs. C]) revealed changes in functional performance specifically in the subgroup with worse structural impairment. **(C)** The lateral prefrontal cortex showed such an association specifically to the adaptive subgroup (i.e., subgroup B) but not in the non-adaptive group (i.e., subgroup D) or the other subgroups. The findings strengthen the role that an increased gray matter volume at the prefrontal cortex, which may imply a better individual brain reserve in cognitive processing and learning, would contribute to better adaptation in masticatory performance. ^∗^The functional performance of the adaptive subgroup.

We first clarify the association between functional performance and challenges. In our case, we adopted a between-group design, and the sample was subgrouped by the degree of structural impairment based on the Eichner Index, which reflects the degree of posterior contact, and the masticatory performance (Figure 2A). The subgroup with fewer teeth contact would have a lower masticatory performance, based on the previous findings (Ikebe et al., 2012). The task of assessing functional performance (gum-chewing) was standardized for both groups.

#### Equalize the Oromotor Performance for Comparison

Our next step is to equalize the functional performance between the subgroups. Because we aimed to understand how masticatory function is maintained in the condition of structural impairment, each group was further subgrouped by masticatory performance via median split (Figure 2A). Based on the subgrouping, subgroup B represents the subjects who had fewer teeth contact but maintained a good masticatory performance (i.e., the adapted group) that showed no statistically significant difference from subgroup A (Figure 2B). Subgroup D, which represents the subjects who did not adapt to the loss of posterior contact, would show a significantly lower masticatory performance, compared to either subgroup A or B. Notably, what we are interested in is the brain signature that will reflect the interaction between the structural and functional factors. The contrast between subgroups A and B or between subgroups C and D only revealed the changes explained by structural impairment. In contrast, the interactional effect ([B vs. D] vs. [A vs. C]) revealed changes in functional performance specifically in the subgroup with worse structural impairment (Figure 2B). For example, in the current case, the gray matter volume at the lateral prefrontal cortex reflected the interactional effect of functional adaptation (Figure 2B).

#### Focus on the Individual Differences in Brain/Cognitive Reserve

If the brain signature is associated with functional adaptation, we may expect that variations in this signature would explain individual variations in functional adaptation. Critically, since the brain signature specifically reflects individual differences in performance, a significant correlation would be identified only in the subgroup showing adaptation (i.e., subgroup B) but not in the other subgroups (Figure 2C). In this case, the lateral prefrontal cortex showed such an association specifically to the adaptive subgroup (i.e., subgroup B) but not in the non-adaptive group (i.e., subgroup D) or the other subgroups. The findings strengthen the role that an increased gray matter volume at the prefrontal cortex, which may imply a better individual brain reserve in cognitive processing and learning, would contribute to better adaptation in masticatory performance.

### Statistical Considerations

The hypothetical experimental framework should be investigated with careful considerations from research design and statistical analysis. First of all, the example that was proposed previously is a cross-sectional observational research. It may help to identify the brain region associated with the individual variations in adaptation. However, it does not disclose the dynamic process of functional adaptation, which should be identified through a longitudinal observation. Secondly, either for a cross-sectional or a longitudinal design, the independent and dependent variables and potentially confounding factors need to be clarified. For example, when it comes to *what functional performance is regained*, one should clarify how masticatory performance is assessed: a self-report of chewing experience or the results from objective assessment (e.g., oral mixing tests or cutting/crunching tests). Third, all the observed variables may covariate with some confounding factors. For example, an increased degree of structural impaired, such as tooth loss, may be associated with orofacial pain. And some general factors, such as general physical ability (e.g., grip force) and the use of medication may be associated with oral functions (Morita et al., 2018; Yamaguchi et al., 2018). These factors should be carefully considered in the statistical model. Finally, it should be noted that either the within-subject or between-group comparison should be interpreted on the basis of an adequate statistical power and a proper estimation of effect size. The under-powered results suffer from an increased risk of type II error. The lack of adequate statistical power may be associated with a small sample size, which is not uncommon in neuroimaging research (Button et al., 2013). The estimation of the training-related effect size is particularly critical from the clinical perspective. A task that leads to a small effect size – even being statistically significant – would still be clinically insignificant.

## Practical Implications and Further Considerations

### Implications for Geriatric Patients With Neurological Disorders

There is an urgent demand for dentists to focus on geriatric patients with neurological disorders, including dementia and stroke. These disorders have posed a great challenge to clinical management because they may interfere with regular dental assessments or therapies, which successfully work in healthy older patients. For example, dysphagia remains a huge challenge in patients with dementia (Boccardi et al., 2016). Notably, patients with dementia have a problem with the cognitive aspects of swallowing. For example, they may require a cue from the other person to initiate eating (Priefer and Robbins, 1997). The score from the Mini-Mental State Examination was inversely associated with the suspected rate of aspiration (Rosler et al., 2015). Even when they receive training in swallowing, they may be less able to follow these instructions and keep doing them regularly (Wirth et al., 2016). The recent findings regarding cognitive processing and motor learning of oromotor functions can provide a better evaluation of the oral sensorimotor functions, which could be pivotal to formulate evidence-based clinical management of geriatric and special needs patients.

### Implications for the Neuroplasticity of Oral Rehabilitation

Evidence from animal research has revealed that the sensorimotor cortices show a plastic effect that responds to changes in oral functions (Avivi-Arber et al., 2011; Avivi-Arber and Sessle, 2018). In mice, a widespread change in the volumes of multiple cortical brain regions, including the areas associated with sensorimotor, cognitive and emotional functions, were identified, following the extraction of molar teeth (Avivi-Arber et al., 2017). Brain plasticity can also be identified in human subjects with oromotor training or prosthetic treatment (Kumar et al., 2018). It should be noted that this effect implies a cause-effect relationship, i.e., the changes in brain capacity respond to experienced demands (Lindenberger et al., 2017). However, most of the cross-sectional neuroimaging studies primarily revealed correlational but not causal results (Poldrack and Farah, 2015). Nevertheless, these cross-sectional findings from human subjects would be valuable for further animal and neuroimaging research based on intervention. When a sensorimotor intervention is adopted to elucidate the cause-effect relationship of learning and brain plasticity, the protocol of the intervention should be clearly defined. For example, the intervention can be a tactile stimulus (e.g., repetitive sensory stimulation), which improved tactile performance and revealed a corresponding plastic effect on brain structure and intrinsic functional network (Heba et al., 2017; Schmidt-Wilcke et al., 2018). In terms of orofacial research, the intervention can be a standardized training protocol, e.g., the food biting task (Kumar et al., 2019), or a pre- vs. post-treatment comparison of denture installation (Luraschi et al., 2013). Notably, either repetitive stimulation or the use of denture has revealed brain plasticity at the somatosensory region (Luraschi et al., 2013; Heba et al., 2017; Schmidt-Wilcke et al., 2018). Such a convergent finding would strengthen the role of sensory feedback in adaptation in sensorimotor functions, helping to clarify the cause-effect relationship between learning and plasticity.

### Implications for Geriatric Patients With Normal Aging

It is noteworthy that functional adaptation would be a general issue for all geriatric dental patients, not just for those with severe physical/cognitive impairment. Indeed, most elderly patients were satisfied with their dentures (Carlsson and Omar, 2010), and through the advent of implant dentistry, masticatory functions can be substantially improved (for a detailed review, see Trulsson et al., 2012). However, there are still pronounced individual differences in their experience of improvement. For example, a randomized controlled trial revealed that an implant-supported overdenture, compared to the denture with conventional relines, improved one’s maximum bite force. However, the scores of masticatory performance and the nutritional scale did not show a significant difference between the two therapies (Muller et al., 2013). In elderly people of Eichner Index C (i.e., without occlusal contact), a great variation in masticatory performance was shown (Ikebe et al., 2012). The clinical findings imply that even under the same condition of structural impairment (e.g., being edentulous), older people may adapt to this challenge to different degrees.

## Conclusion

This Focused Review highlighted that the functional aspects of adaptation—which would be predominantly associated with the brain mechanisms of cognitive processing and motor learning—play a critical role in the individual differences in the adaptive behaviors of oromotor functions. Issues about how individuals acquire new oral sensorimotor skills and the mechanisms underlying the individual differences in adaptation require further investigation. Understanding the brain-stomatognathic mechanisms underlying sensorimotor adaptation may provide important insight into the age-related changes in oral functions and contribute to the clinical management of dental patients.

## Author Contributions

C-SL conceived and wrote the review.

## Conflict of Interest

The author declares that the research was conducted in the absence of any commercial or financial relationships that could be construed as a potential conflict of interest.

## Footnotes

1 *Medical Subject Headings* (Online). Available at: https://www.ncbi.nlm.nih.gov/mesh/68000375 (accessed August 12, 2019).
